# Clinical evidence for respiratory insufficiency type II predicts weaning failure in long-term ventilated, tracheotomised patients: a retrospective analysis

**DOI:** 10.1186/s40560-018-0338-0

**Published:** 2018-10-16

**Authors:** Friederike Sophie Magnet, Hannah Bleichroth, Sophie Emilia Huttmann, Jens Callegari, Sarah Bettina Schwarz, Claudia Schmoor, Wolfram Windisch, Jan Hendrik Storre

**Affiliations:** 1 Cologne Merheim Hospital, Department of Pneumology, Faculty of Health/School of Medicine, Kliniken der Stadt Köln gGmbH, Witten/Herdecke University, Cologne, 51109 Germany; 2 Department of General, Visceral and Vascular Surgery, St.-Josefs-Hospital Freiburg, Freiburg im Breisgau, 79104 Germany; 3 Clinical Trials Unit, Faculty of Medicine and Medical Center, University of Freiburg, Freiburg im Breisgau, 79106 Germany; 4 Department of Intensive Care, Sleep Medicine and Mechanical Ventilation, Asklepios Fachkliniken Munich-Gauting, Robert-Koch-Allee 2, 82131 Gauting, Germany; 5 Department of Pneumology, University Medical Hospital, Freiburg im Breisgau, 79106 Germany

**Keywords:** Mechanical ventilation, COPD, Weaning, Tracheostomy, Respiratory failure

## Abstract

**Background:**

Patients who require a prolonged weaning process comprise a highly heterogeneous group of patients amongst whom the outcome differs significantly. The present study aimed to identify the factors that predict whether the outcome for prolonged weaning will be successful or unsuccessful.

**Methods:**

Data from tracheotomised patients who underwent prolonged weaning on a specialised weaning unit were assessed retrospectively via an electronic and paper-bound patient chart. Factors for weaning success were analysed by univariate and multivariate analyses.

**Results:**

Out of the 124 patients examined, 48.4% were successfully weaned (*n* = 60). Univariate analysis revealed that long-term home mechanical ventilation prior to current weaning episode; time between intubation and the first spontaneous breathing trial (SBT); time between intubation and the first SBT of less than 30 days; lower PaCO_2_ prior to, and at the end of, the first SBT; and lower pH values at the end of the first SBT were predictors for successful weaning. Following multivariate analysis, the absence of home mechanical ventilation prior to admission, a maximum time period of 30 days between intubation and the first SBT, and a non-hypercapnic PaCO_2_ value at the end of the first SBT were predictive of successful weaning.

**Conclusions:**

The current analysis demonstrates that the evidence for respiratory insufficiency type II provided by clinical findings serves as a predictor of weaning failure.

## Background

Weaning a patient off mechanical ventilation (MV) plays a key role in modern pneumology and long-term mechanical ventilation [1]. Three different categories of weaning have been established by Boles and colleagues: category 1: simple weaning, category 2: difficult weaning, and category 3: prolonged weaning [1]. These categories are defined according to the time and number of unsuccessful spontaneous breathing trials (SBTs) the patient needs (after the first unsuccessful SBT) until he/she can be extubated; this process occurs under the condition of readiness to wean [1]. Here, prolonged weaning is defined as failure of at least three weaning attempts or requiring of > 7 days of weaning after the first SBT [1].

According to literature, 30–60% of patients are classified into the simple weaning category, 25–40% into the difficult weaning category, and 6–30% into the prolonged weaning category [2–5]. A new definition for weaning categories has recently been proposed, based on the fact that not all weaning patients can be classified according to the Boles criteria, for example, those who never underwent an SBT (i.e. successful self-extubation, no weaning attempt possible) [6]. For this purpose, the new classification is based on the amount of time required by intubated or tracheotomised patients to achieve weaning success or separation from mechanical ventilation, respectively, following the first separation attempt. This particular classification also defines prolonged weaning patients (category 3) as either successful or unsuccessful [6]. However, this new classification requires further evaluation.

Patients classified into the prolonged weaning group based on the Boles criteria form a highly heterogeneous group of patients. This is due to extremely different underlying conditions, differences in the leading cause of respiratory insufficiency type II, various underlying reasons for acute respiratory failure, and significant variation in the occurrence of co-morbidities that contribute to respiratory failure [7]. Since the outcome of weaning success differs considerably in these patients, it would be desirable to assess in advance whether a patient undergoing a prolonged weaning process (category 3) can ultimately be successfully weaned or not. To date, the evaluation of predictors for weaning success/failure in prolonged weaning patients has not been performed; therefore, the present study aimed to identify these factors.

## Methods

### Patients

Patient data collected between January 2009 and December 2011 were assessed retrospectively via electronic and paper-bound patient charts. All data were derived from patients who were treated in the weaning unit at the Department of Pneumology, Lung Clinic, Cologne Merheim Hospital, Witten/Herdecke University, Germany. The local ethics committee ruled that no formal ethics approval was required in this particular case. Patients were included if they were already invasively ventilated via tracheostomy upon admission to the weaning unit and if they had at least one documented SBT whilst in the weaning unit. Orotracheally intubated patients were not included. All patients were admitted to the unit to identify and carry out the weaning process. They were either transferred to our weaning unit from an external hospital, from an external hospital via our own (=internal) ICU, from our internal ICU (after direct admission from the emergency department at our internal ICU), from the general ward, from a facility for long-term MV, or from the domestic setting if invasive home mechanical ventilation (HMV) had been established before.

Patients allocated to the simple and difficult weaning categories according to Boles et al. [1] were excluded from the study. If patients underwent weaning stints more than once during the observation period, only the first episode was analysed. Weaning was systematically performed according to the recommendations from the statement by Boles and colleagues [1] as well as to national guidelines on prolonged weaning [8]. Here, SBTs were performed using T-piece with successful SBT being defined according to the Boles criteria [1].

Patients were discharged from the weaning unit if weaning was successful or if weaning was suggested to be unsuccessful; here, unsuccessful weaning was established by the specialised treatment team including the senior physician.

### Data analysis

The following data were assessed for each patient: demographic data; diagnosis at admission; most likely cause of respiratory failure according to medical history; pre-existing HMV and long-term oxygen therapy (LTOT); date of intubation; the first SBT/extubation or interruption to weaning; length of time between intubation and the first SBT (days); length of time between admission and the first SBT (days); duration of the first SBT (min), duration of weaning (days between the first SBT and extubation/weaning interruption); duration of hospital stay in the weaning unit (days); use of non-invasive ventilation (NIV) during weaning; arterialized blood gas analysis (earlobe) at admission (arterialized partial pressure of carbon dioxide (PaCO_2_)), and before, and at the time of termination of the first SBT (=‘after the first SBT’); arterialized partial pressure of oxygen (PaO_2_), PaCO_2_, pH); and haemoglobin (g/dl) at the time of the first SBT; as well as base excess (BE) and standard bicarbonate (HCO_3_^−^) at admission. Patients were classified at the end of the weaning period into either successful weaning (with or without NIV) or unsuccessful weaning (death or discharge with invasive ventilation) categories according to Boles et al. [1].

### Statistical analysis

The effects of the following factors on weaning success were analysed: age (continuous, change per 10 years); sex (male, female); number of known previous diseases at time of admission (continuous, change per 1 disease); coronary artery disease (yes, no); congestive heart failure (yes, no); leading cause of respiratory insufficiency (pneumonia, sepsis, COPD, other); long-term HMV prior to current weaning episode (yes, no); time between intubation and the first SBT (cut-off ≤ 14 days and > 14 days as well as ≤ 30 days and > 30 days); length of time between admission to the weaning unit and the first SBT (continuous, change per 1 day); duration of the first SBT (cut-off of ≤ 60 min vs. > 60 min); PaCO_2_ > 45 mmHg at admission (yes, no); PaCO_2_ before the first SBT (continuous, change per 10 mmHg); PaCO_2_ > 45 mmHg before the first SBT (yes, no); pH before the first SBT (continuous, change per 0.1 units); PaO_2_ before the first SBT (continuous, change per 10 mmHg); PaCO_2_ after the first SBT (continuous, change per 10 mmHg); pH after the first SBT (continuous, change per 0.1 units); and PaO_2_ after the first SBT (continuous, change per 10 mmHg). Categorisation of factors was pre-specified without looking at the data. For the descriptive analysis, continuous data were summarised by arithmetic mean, standard deviation, minimum, 25% quantile, median, 75% quantile, maximum, and the number of complete and missing observations. Categorical data were summarised using the total number of patients in each category, the number of missing values, and the relative frequencies, the latter of which were determined by dividing the number of patients in each category by the total number of patients with non-missing values.

The prognostic effect of the factors on weaning outcome was analysed with logistic regression models. Odds ratios with 95% confidence intervals were calculated, and the effects were tested using the Wald tests. The factors were first analysed separately in univariate analyses. Factors showing an effect with *p* < 0.05 in the univariate analyses were next analysed simultaneously using multiple logistic regression models. One multiple regression model only included baseline factors before the first SBT, whilst another multiple regression model included baseline factors before the first SBT, as well as factors measured after the first SBT simultaneously. All analyses were exploratory, without former sample size planning. No adjustment for multiple testing was performed. All *p* values should be interpreted in a descriptive sense. All analyses were performed with Statistical Analysis System (SAS 9.2).

## Results

**Fig. 1:**
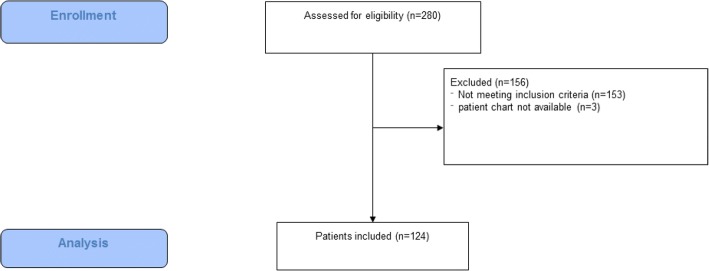
CONSORT flow chart of assessed patient charts

Figure 1 presents the CONSORT flow diagram summarising the patient enrolment/assessment procedure (online data supplement). A total of 124 patients were included the analysis.

The basic demographic data from the whole cohort were as follows: 41.1% (*n* = 51) were female; median age was 71 years (interquartile range (IQR) 62–78 years); median BMI was 25.7 kg/m^2^ (IQR 21.6–33.1 kg/m^2^); and median count of known diagnoses at the time of admission was 5 (IQR 4–6), with most of the known diagnoses at admission being related to the respiratory system (*n* = 118), followed by the cardiac system (*n* = 90). Concerning metabolic status, median BE was 8.2 mmol/l (IQR 4.8–11.7 mmol/l) and median HCO_3_^−^ was 31.7 mmol/l (IQR 28.8–34.1 mmol/l).

Further baseline characteristics are presented in Table 1.

**Table 1:** Baseline characteristics of the study cohort

Factor	Weaning success *n* = 60	Weaning failure *n* = 64
Smoking status, *n* (%)		
Active smokers at hospital admission	24 (60%)	16 (40%)
Non-/ex-smokers	18 (44%)	23 (56%)
Missing	18	25
Transfer from ..., *n* (%)		
External hospital	24 (50%)	24 (50%)
External hospital via internal ICU	17 (74%)	6 (26%)
Internal ICU	16 (43%)	21 (57%)
General ward	1 (25%)	3 (75%)
Facility for long-term MV	0 (0%)	2 (67%)
Domestic setting	1 (11%)	8 (89%)
LTOT prior to current weaning episode, *n* (%)	2 (11%)	17 (89%)

Data are expressed as *n* (% according to weaning subgroup)

*ICU* intensive care unit, *LTOT* long-term oxygen therapy, *MV* mechanical ventilation

Most patients (*N* = 97; 78.2%) had undergone prolonged weaning after being intubated and tracheotomised during treatment for acute respiratory failure, without a history of long-term ventilation prior to ICU treatment. Eleven (8.9%) patients had been on long-term NIV prior to acute respiratory failure followed by tracheostomy and underwent subsequent prolonged weaning, whilst 16 patients (12.9%) had been on long-term invasive ventilation and were submitted for re-evaluation of weaning following weaning failure prior to submission. Time courses of the different steps within the weaning process are given in Table 2.

**Table 2:** Time courses of the different steps within the weaning process

	Median	IQR
Time between intubation and the first SBT (days)	31	18–54
Duration of the first SBT for successful weaning (min)	60	30–120
Duration of the first SBT for unsuccessful weaning (min)	43	20–120
Duration of weaning after the first SBT for successful weaning (days)	18	10–26
Duration of weaning after the first SBT for unsuccessful weaning (days)	19	7–39

Weaning success rates at the time of hospital discharge were as follows: 48.4% were successfully weaned (*n* = 60), whereas 51.6% had not been successfully weaned by the time of discharge (*n* = 64).

Patient statuses at discharge as well as in-hospital mortality according to weaning outcome are shown in Table 3.

**Table 3:** Patient statuses at discharge and in-hospital mortality

	Weaning success *n* = 60	Weaning failure *n* = 64	*p* value
Therapeutic goal, *n* (%)			0.013
Limitation of therapy	1 (11%)	8 (89%)	
Withdrawal of therapy	0 (0%)	3 (100%)	
In-hospital mortality, *n* (%)	5 (20%)	20 (80%)	0.001
HMV at discharge, *n* (%)			0.000
No ventilation	27 (100%)	0 (0%)	
Invasive HMV	0 (0%)	44 (100%)	
Non-invasive HMV	28 (100%)	0 (0%)	

*HMV* home mechanical ventilation

### Univariate analysis

Patient characteristics, leading cause of respiratory failure, and the type of long-term ventilation prior to the current weaning episode assessed as weaning predictors are presented in Table 4.

**Table 4:** Assessment of patient characteristics, leading cause of respiratory failure, and long-term ventilation history as weaning predictors

Factor	Weaning success *n* = 60	Weaning failure *n* = 64	OR [95% KI]	*p* value
Age (years)				
Mean ± SD	68 ± 12	69 ± 12	0.94 [0.70, 1.28]	0.71
Median (IQR)	69 (61–77)	71 (62–78)		
Min–max	31–85	39–88		
Gender, *n* (%)				
Female	28 (55%)	23 (45%)	1	0.23
Male	32 (44%)	41 (56%)	0.64 [0.31, 1.32]	
Leading cause of respiratory insufficiency, *n* (%)				
AECOPD	22 (42%)	30 (58%)	1	0.38
Pneumonia	18 (62%)	11 (38%)	2.23 [0.88, 5.66]	
Sepsis	8 (50%)	8 (50%)	1.36 [0.44, 4.20]	
Other	12 (44%)	15 (56%)	1.09 [0.43, 2.79]	
Long-term HMV prior to current weaning episode, *n* (%)				
None	56 (58%)	41 (42%)	1	0.0004
Yes	4 (15%)	23 (85%)	0.13 [0.04, 0.40]	

Data are expressed as *n* (% according to weaning subgroup), mean ± SD, median (IQR), min–max, as well as missing data (missing)

*ALI* acute lung injury, *ARDS* acute respiratory distress syndrome, *CHF* congestive heart failure, *AECOPD* acute exacerbated chronic obstructive pulmonary disease, *IQR* interquartile range, *HMV* home mechanical ventilation, *max* maximum value, *min* minimum value, *NIV* non-invasive ventilation, *SD* standard deviation

There were no detectable effects of congestive heart failure (OR 1.10 [0.52, 2.34], *p* = 0.80) or coronary artery disease (OR 1.87 [0.87, 4.02], *p* = 0.11) on the probability of weaning success.

The mean length of time between intubation and the start of the weaning process is summarised with respect to the final weaning status in Table 5.

**Table 5:** Length of time between intubation and the first spontaneous breathing trial summarised with respect to weaning outcome

Factor	Weaning success *n* = 60	Weaning failure *n* = 64	OR [95% KI]	*p* value
Time (days) between intubation and 1st SBT				
Mean ± SD	31 ± 26	192 ± 484		
Median (IQR)	23 (17–36)	40(19–89)		
Min–max	2–143	2–2730		
Missing	1	1		
*n* (%)				
> 30 days	21 (34%)	41 (66%)	1	0.001
≤ 30 days	38 (63%)	22 (37%)	3.37 [1.60, 7.09]	

Data are expressed as *n* (% according to the underlying weaning subgroup), mean ± SD (min–max), median (IQR) as well as missing data (missing)

*IQR* interquartile range, *max* maximum value, *min* minimal value, *MV* mechanical ventilation, *SBT* spontaneous breathing trial, *SD* standard deviation

Patients who needed less than 30 days had a higher rate of weaning success, but no effect of ‘time between intubation and the first SBT’ was shown when a cut-off of 14 days was used (OR 0.60 [0.23, 1.58], *p* = 0.30). Similarly, there was no effect of ‘time between weaning unit admission and the first SBT’ on weaning success (OR 1.00 [0.83, 1.22], *p* = 0.98).

The median duration of the first SBT was 60 min (IQR 30–120) in successfully weaned patients compared to 43 min (IQR 20–120) in unsuccessfully weaned patients. Accordingly, the univariate analysis showed no difference with regard to the weaning success between patients with a shorter vs. longer duration of the first SBT (cut-off ≤ 60 min vs. > 60 min; OR 0.74 [0.36, 1.55], *p* = 0.43).

Table 6 contains the blood gas analysis during the SBT. These values demonstrate an impact on the weaning success rate of PaCO_2_ prior to, and at the end of, the first SBT, and of pH values at the end of the first SBT. In contrast, pH values prior to the first SBT, PaO_2_ values prior to the first SBT, and PaO_2_ values at the end of the first SBT did not show an effect on weaning when a significance level of 0.05 was set.

**Table 6:** Blood gas analysis during the SBT

Factor	Weaning success *n* = 60	Weaning failure *n* = 64	OR [95% KI]	*p* value
PaCO_2_ > 45 mmHg at admission, *n* (%)				
No	35 (53%)	31 (47%)	1	0.23
Yes	24 (42%)	33 (58%)	0.64 [0.32, 1.32]	
Missing	1	0		
PaCO_2_ prior to the first SBT (mmHg)				
Mean ± SD	40 ± 7	45 ± 14	0.61 [0.41, 0.93]	0.021
Median (IQR)	40 (36–43)	44 (36–54)		
Min–max	18–59	23–115		
Missing	1	1		
PaCO_2_ > 45 mmHg prior to the first SBT, *n* (%)				
No	50 (56%)	39 (44%)	1	0.006
Yes	9 (27%)	24 (73%)	0.29 [0.12, 0.70]	
PaCO_2_ after the first SBT (mmHg)				
Mean ± SD	45 ± 9	55 ± 12	0.40 [0.26, 0.62]	< 0.0001
Median (IQR)	43 (39–48)	54 (47–63)		
Min–max	32–81	29–89		
Missing	2	10		
pH values after the first SBT				
Mean ± SD	7.46 ± 0.05	7.41 ± 0.09	3.23 [1.66, 6.31]	0.0006
Median (IQR)	7.46 (7.44–7.49)	7.42 (7.36–7.46)		
Min–max	7.36–7.56	7.19–7.67		
Missing	3	10		
pH values prior to the first SBT				
Mean ± SD	7.50 ± 0.06	7.49 ± 0.09	1.15 [0.71, 1.86]	0.58
Median (IQR)	7.49 (7.46–7.54)	7.49 (7.42–7.56)		
Min–max	7.40–7.68	7.26–7.68		
Missing	1	1		
PaO_2_ prior to the first SBT (mmHg)				
Mean ± SD	74 ± 19	80 ± 30	0.91 [0.78, 1.06]	0.22
Median (IQR)	75 (61–87)	72 (60–97)		
Min–max	34–115	37–169		
Missing	3	5		
PaO_2_ values at the end of the first SBT				
Mean ± SD	71 ± 24	85 ± 41	0.88 [0.77, 1.00]	0.056
Median (IQR)	65 (52–79)	70 (62–97)		
Min–max	42–139	32–231		
Missing	6	13		

Data are expressed as *n* % according to the underlying weaning subgroup), mean ± SD (min–max), median (IQR) as well as missing data (missing)

*IQR* interquartile range, *max* maximum value, *min* minimal value, *PaCO*_*2*_ arterial partial pressure of carbon dioxide, *PaO*_*2*_ arterial partial pressure of oxygen, *SD* standard deviation

### Multivariate analysis

**Fig. 2:**
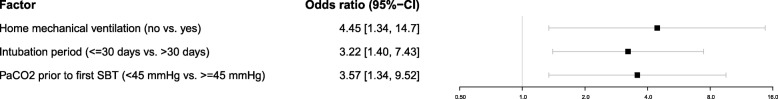
Multivariate analysis of baseline factors for weaning success vs. failure. PaCO_2_ = partial pressure of carbon dioxide, SBT = spontaneous breathing trial

Multivariate analysis of baseline factors for weaning success vs. failure revealed that the likelihood of successful weaning was increased when there was an absence of HMV (either invasive or non-invasive) prior to admission (*p* = 0.015), an intubation period of ≤ 30 days (*p* = 0.006), and a PaCO_2_ value < 45 mmHg prior to the first SBT (*p* = 0.011) (Fig. 2).

**Fig. 3:**
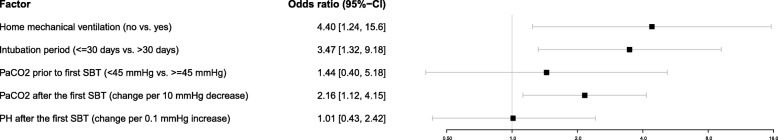
Joint multivariate analysis of baseline factors and post-first SBT factors. PaCO_2_ = partial pressure of carbon dioxide, SBT = spontaneous breathing trial

A joint analysis of baseline factors and post-first SBT factors demonstrated that a lower PaCO_2_ value after the first SBT rather than before was more important for weaning success. A lower PaCO_2_ value after the first SBT (change per 10 mmHg decrease) increased the likelihood of weaning success vs. failure (*p* = 0.021), whereas PaCO_2_ levels prior to the first SBT had no effect when PaCO_2_ was included in the analysis, as illustrated in Fig. 3.

## Discussion

This is the first study to evaluate candidate parameters as outcome predictors of weaning in patients undergoing prolonged weaning (as defined by international consensus criteria [1]) from invasive mechanical ventilation via tracheal cannulae. The main findings were that the following factors are predictive of successful weaning: (1) absence of home mechanical ventilation prior to admission, (2) maximum duration of 30 days between intubation and the first SBT, and (3) a non-hypercapnic PaCO_2_ value at the end of the first SBT.

It is well established that weaning failure is associated with respiratory insufficiency type II [9–11]. Recent trials suggest that non-invasive detection of diaphragmatic dysfunction via ultrasound in patients undergoing a difficult or prolonged weaning process may predict weaning failure [12]. This also reportedly holds true for a longer stay on the intensive care unit [13]. Therefore, it appears plausible that patients who also present with clinical evidence for respiratory insufficiency type II have a higher risk of weaning failure than patients without clinical evidence for respiratory insufficiency type II. Most importantly, this pertains to patients who had pre-existing HMV therapy, since these patients were already treated for respiratory insufficiency type II prior to acute respiratory failure. Here, low PaCO_2_ values would hint at a sufficient respiratory pump function. The current data further supports that pre-existing HMV is an independent risk factor for unsuccessful weaning, apart from time of intubation until beginning of weaning. Furthermore, it is known that prolonged mechanical ventilation can lead to diaphragmatic atrophy and contractile dysfunction—known as ventilator-induced diaphragmatic dysfunction [14–16]—and that these changes are related to the duration of mechanical ventilation [15]. Moreover, long-term mechanical ventilation is associated with a higher rate of morbidity and mortality [17]. Taken together, these aspects support our observation that a shorter time period between intubation and the first SBT is in favour of a successful weaning outcome.

Prolonged mechanical ventilation can range from 14 [7] to 21 [18] days, according to different definitions. Based on this, two different time periods occurring between intubation and the first SBT (cut-off of 14 and 30 days) were chosen for analysis in the current study. It was found that mechanical ventilation was associated with a higher rate of weaning success in patients who underwent shorter periods of mechanical ventilation, albeit only when the cut-off was set at 30 days. This is likely explained by the fact that patients did not begin the weaning process during the first 14 days because during this time, it was mainly necessary to treat acute respiratory failure due to the severity of the underlying disease. This is also supported by a study showing that patients who were admitted to a respiratory ICU in 2005 generally were more severely ill than they used to compared in 1991 and that the clinical outcome of these patients has worsened over this time period [19].

Invasive HMV is of great socio-economic burden. In addition, recent trials have demonstrated that health-related quality of life can be severely impaired in patients receiving invasive HMV following weaning failure [20, 21]. As shown by the current trial, the duration of invasive mechanical ventilation predicts weaning success. Therefore, ICU medicine should focus on avoiding prolonged mechanical ventilation and subsequent prolonged weaning. This requires modern treatment strategies including extra-corporal CO_2_-removal aimed at avoiding intubation or at reducing the duration of invasive mechanical ventilation [22]. Furthermore, end-of-life decision making is also suggested to be important with regard to this discussion [8].

There are some limitations of the study which need to be addressed. Firstly, the study was retrospectively designed without sample size pre-planning; therefore, all results have to be interpreted in an exploratory context and require further prospective evaluation. Secondly, this is a single-centre study that only included tracheotomised (but not intubated) patients who were primarily non-surgical patients with underlying respiratory diseases. Therefore, the current analysis is only valid for this particular patient group and should be interpreted carefully if transferred to other patient groups undergoing prolonged weaning.

## Conclusions

In conclusion, this retrospective study revealed that in patients with prolonged weaning, successful weaning was associated with the absence of home mechanical ventilation established prior to acute respiratory failure, with a shorter period of mechanical ventilation, and lower PaCO_2_ values after the first SBT. Therefore, the current analysis supports the notion that respiratory insufficiency type II that is defined by clinical findings is predictive of weaning failure.

## Abbreviations

AECOPD: Acute exacerbated chronic obstructive pulmonary disease
ALI: Acute lung injury
ARDS: Acute respiratory distress syndrome
CHF: Congestive heart failure
CI: Confidence interval
HMV: Home mechanical ventilation
ICU: Intensive care unit
IQR: Interquartile range
LTOT: Long-term oxygen therapy
Max: Maximum value
Min: Minimum value
OR: Odds ratio
PaCO_2_: Arterialized partial pressure of carbon dioxide
PaO_2_: Arterialized partial pressure of oxygen
SD: Standard deviation
